# Effects of low and high tidal volume and pentoxifylline on intestinal blood flow and leukocyte-endothelial interactions in mechanically ventilated rats

**DOI:** 10.1186/cc10723

**Published:** 2012-03-20

**Authors:** N Nakagawa, P Aikawa, HZ Zhang, C Correia, R Pazzeti, C Valente Barbas, T Mauad, E Silva, P Sannomiya

**Affiliations:** 1 Faculdade de Medicina da Universidade de São Paulo, Brazil; 2 University of Toronto and Saint Michael Hospital, Toronto, Canada

## Introduction

The combination of high positive end-expiratory pressure (PEEP) and low tidal volume (VT) decreases some risks of mechanical ventilation, including pulmonary overdistention, damage due to cyclic opening and closing of the alveoli, and inflammatory responses that can lead to multiple-organ dysfunction. We hypothesized that high VT and high PEEP induce mesenteric microcirculatory disturbances and that those disturbances would be attenuated by pentoxifylline, which is anti-inflammatory.

## Methods

We anesthetized (isoflurane 1.5%), tracheostomized, and mechanically ventilated 57 male Wistar rats with PEEP of 10 cmH_2_O and FIO_2 _of 0.21 for 2 hours. One group received low VT (7 ml/kg), another group received high VT (10 ml/kg), and a third group received high VT (25 mg/kg) plus pentoxifylline. We measured mean arterial pressure, respiratory mechanics, mesenteric blood flow, and leukocyte-endothelial interactions.

## Results

The mean arterial pressure was similar among the groups at baseline (108 mmHg (IQR 94 to 118 mmHg)) and after 2 hours of mechanical ventilation (104 mmHg (IQR 90 to 114 mmHg)). Mesenteric blood flow was also similar between the groups: low VT 15.1 ml/minute (IQR 12.4 to 17.7 ml/minute), high VT 11.3 ml/minute (IQR 8.6 to 13.8 ml/minute), high-VT/pentoxifylline 12.4 ml/minute (10.8 to 13.7 ml/minute). Peak airway pressure was lower (*P *= 0.03) in the low-VT group (10.4 cmH_2_O (IQR 10.2 to 10.4 cmH_2_O)) than in the high-VT group (12.6 cmH_2_O (10.2 to 14.9 cmH_2_O)) or the high-VT/pentoxifylline group (12.7 cmH_2_O (10.7 to 16.0 cmH_2_O)). There were fewer adherent leukocytes (*P *= 0.005) and fewer migrated leukocytes (*P *= 0.002) in the low-VT group (5 cells/100 μm length (IQR 4 to 7 cells/100 μm length) and 1 cell/5,000 μm^2 ^(IQR 1 to 2 cells/5,000 μm^2^), respectively) and the high-VT/pentoxifylline group (5 cells/100 μm length (IQR 3 to 10 cells/100 μm length) and 1 cell/5,000 μm^2 ^(IQR 1 to 3 cells/5,000 μm^2^), respectively) than in the high-VT group (14 cells/100 μm length (IQR 11 to 16 cells/100 μm length) and 9 cells/5,000 μm^2 ^(IQR 8 to 12 cells/5,000 μm^2^), respectively).

## Conclusion

Low VT with high PEEP was lung-protective, and early pentoxifylline reduced the inflammatory response to high VT with high PEEP (and presumed lung overdistention) during mechanical ventilation.

